# Investigation of the Relationship Between Type D Personality and Depression, Anxiety and Somatosensory Amplification in Patients With Fibromyalgia

**DOI:** 10.1155/prm/5315083

**Published:** 2025-05-15

**Authors:** Meltem Hazel Şimşek, Ulaş Korkmaz, Fatma Gül Helvacı Çelik, Nurçe Çilesizoğlu Yavuz, Çiçek Hocaoğlu

**Affiliations:** ^1^Faculty of Medicine, Department of Psychiatry, Giresun University, Giresun, Turkey; ^2^Faculty of Medicine, Department of Physical Medicine and Rehabilitation, Giresun University, Giresun, Turkey; ^3^Faculty of Medicine, Department of Psychiatry, Recep Tayyip Erdogan University, Rize, Turkey

**Keywords:** anxiety, depression, fibromyalgia, psychosomatic disorders, somatosensory amplification, type D personality

## Abstract

**Objective:** This study aimed to investigate differences in depression, anxiety and somatosensory amplification between fibromyalgia (FM) patients with and without type D personality (TDP) and healthy controls and to examine the mediating role of somatosensory amplification in the relationship between TDP and FM severity.

**Methods:** A total of 159 participants were included in the cross-sectional case-control study and divided into three groups: FM patients with TDP (*n* = 56, mean age = 45.93 ± 11.01), FM patients without TDP (*n* = 48, mean age = 49.17 ± 11.18) and healthy controls (*n* = 55, mean age = 46.1 ± 9.64). Participants were assessed with the Fibromyalgia Impact Questionnaire (FIQ; administered only to FM patients), TDP Scale, Beck Depression Inventory, Beck Anxiety Inventory and Somatosensory Amplification Scale. Mediation analysis was performed to determine the mediating role of somatosensory amplification.

**Results:** FM patients with TDP had significantly higher levels of depression, anxiety and somatosensory amplification compared to both FM patients without TDP and healthy controls (*p* < 0.001). Correlation analyses showed strong positive associations between TDP and anxiety (*r* = 0.729, *p* < 0.001) and depression (*r* = 0.794, *p* < 0.001). Somatosensory amplification was found to have a significant mediating role in the relationship between TDP and FM severity (*b* = 0.084, 95% CI = 0.018–0.172, *p* < 0.05).

**Conclusion:** These results highlight TDP as an important psychological risk factor associated with increased depression, anxiety, and somatosensory amplification in FM patients. The apparent mediating role of somatosensory amplification suggests that addressing this mechanism and psychological stress with targeted psychosocial interventions may improve the efficacy of FM treatment.

## 1. Introduction

Fibromyalgia (FM) is a chronic disease that primarily presents with widespread body pain. Patients often describe symptoms such as fatigue, sleep problems, cognitive difficulties and mood disturbances. The disease has a significant impact on daily functioning and quality of life [1, 2]. In addition to widespread pain, FM is often associated with somatic symptoms such as numbness, tingling, gastrointestinal complaints, dizziness, cognitive impairment and dyspnoea [3]. Diagnosis is based on clinical evaluation and symptoms expressed by the patient [3, 4].

In recent studies, the importance of psychological factors has increased due to their influence on the clinic, prognosis and treatment responses of FM. [5]. Personality traits in FM, especially type D personality (TDP), have attracted attention due to their negative impact on the disease [6]. TDP, first defined by Denollet, consists of two basic subcomponents. These are; negative affect and social inhibition [7]. Negative affect refers to negative emotions such as anxiety, depression, irritability and distress. Social inhibition includes difficulty in expressing emotions in social context and avoidance behaviours [8]. TDP has been shown to predispose to psychiatric disorders. It has also been reported to be associated with poor quality of life in various chronic diseases [9–11].

The association of TDP with somatosensory amplification is thought to be effective through negative affect and social inhibition, which are the main components of this personality type. TDP may cause individuals to perceive bodily sensations as threatening. In a study, it was reported that somatosensory amplification levels were significantly higher in individuals with panic disorder and TDP [12]. This suggests that TDP may increase sensitivity to bodily perceptions and thus lead to exacerbation of clinical symptoms.

Personality traits play an important role in FM by influencing symptom perception, mental distress and coping skills [13]. Studies have shown that TDP increases pathological negative affect and social isolation. This reduces psychological resilience and the capacity to enjoy pleasure [14, 15]. It was found that FM patients with TDP experienced more severe pain, had more loss of functionality and had a worse quality of life than FM patients without TDP traits [15].

Another important concept explaining symptom perception in FM is somatosensory amplification. Somatosensory amplification refers to the tendency to perceive bodily sensations as severe, threatening or harmful and to pay excessive attention to them [16, 17]. Somatosensory amplification is quite common in FM patients. This leads to a more intense sensation of pain. It is usually associated with mental distress and decreased functioning [18]. However, research examining the relationship between TDP traits and somatosensory amplification is quite limited.

Anxiety and depression often accompany FM. The accompaniment of psychological factors in FM increases symptom severity (SS) and has a negative effect on the course of the disease [19]. At the same time, TDP accompanying chronic diseases increases the risk of depression and anxiety [20, 21]. However, the mechanisms explaining the relationship between TDP and FM severity have not been fully elucidated.

The main aim of this study was to comprehensively examine the relationship between TDP, somatosensory amplification, depression and anxiety in FM patients. Our primary aim was to determine the relationship between TDP and FM, depression, anxiety and somatosensory amplification levels. The secondary aim is to examine the mediating role of somatosensory amplification between TDP and FM severity.

Our specific hypotheses:

  Hypothesis 1: Depression, anxiety and somatosensory amplification levels are higher in FM patients with TDP than in other groups.
  Hypothesis 2: TDP is positively associated with FM severity and other variables.
  Hypothesis 3: Somatosensory amplification mediates the relationship between TDP and FM severity. This suggests that TDP does not affect FM severity directly, but indirectly through somatosensory amplification.

## 2. Methods

### 2.1. Study Design and Data Source

This study was conducted with a cross-sectional case-control design and the sample size was calculated using G^∗^Power 3.1.9.7 software. Based on previous studies in the literature, the effect size (Cohen's d) was determined as 0.77 and the statistical power as 95%, and it was aimed to reach a total of 135 participants in three groups of 45 people each: FM patients with TDP (*n* = 45), FM patients without TDP (*n* = 45) and healthy controls (HC) (*n* = 45) [22].

Participants were recruited from individuals presenting to the Physical Medicine and Rehabilitation outpatient clinic of a tertiary care hospital between January 2024 and January 2025. Inclusion criteria included being between 18 and 65 years of age and having a diagnosis of FM by a specialist physician according to the 2016 American College of Rheumatology (ACR) criteria [3].

Exclusion criteria include current or lifelong neurological diseases or neurological disability (e.g., epilepsy, multiple sclerosis), inflammatory or rheumatic diseases (e.g., rheumatoid arthritis, lupus), history of active substance abuse within the last 12 months, and major personality disorders (e.g., borderline, antisocial, paranoid, or narcissistic personality disorder) with significant impairment of functioning according to DSM-5 criteria.

These diagnoses were assessed by experienced psychiatrists through clinical observation and semistructured psychiatric interviews based on SCID-5-PD screening questions. Both current and lifetime diagnoses were considered when determining exclusion criteria.

At the end of the evaluation, a total of 104 fibromyalgia patients who were eligible and volunteered to participate in the study were included. 12 patients refused to participate in the study and 18 patients met the exclusion criteria.

The HC group consisted of age and gender matched individuals without a history of fibromyalgia or chronic pain. These individuals did not have TDP characteristics and their eligibility was confirmed by assessments made by a physiatrist and a psychiatrist.

The study protocol was approved by the institutional ethics committee (approval no: 03.07.2024/05) and was performed in accordance with the principles of the Declaration of Helsinki. Written informed consent was obtained from all participants.

### 2.2. Clinical Assessment

Demographic variables include age, education level, occupation, marital status, number of children, and duration of illness.

Clinical interview and data collection process: All interviews and assessments were conducted face-to-face and one-on-one by psychiatry specialists. Interviews were conducted on the day of application or within one week after the application, and each interview lasted 15–20 min on average. Assessment forms were administered to all participants in a quiet and appropriate environment, and necessary guidance was provided to ensure that the forms were filled in correctly and completely.

FM severity in participants was assessed according to the 2016 ACR criteria. This assessment consists of the Widespread Pain Index (WPI) and the SS Scale. The WPI assesses pain points felt at 19 anatomical sites and has a score range of 0–19. The SS assesses fatigue, cognitive complaints and sleep disturbances, with a score range of 0–12. According to these criteria, for the diagnosis of fibromyalgia, WPI ≥ 7 and SS ≥ 5 or WPI = 4–6 and SS ≥ 9 and symptoms should persist for at least three months. Since the scale was developed for clinical diagnosis, internal consistency coefficients (e.g., Cronbach's alpha) were not calculated in the classical sense; however, its validity in clinical diagnosis and severity assessment has been proven and is widely used [3].

Depression levels were measured with the Beck Depression Inventory (BDI), originally developed by Beck et al. and adapted into Turkish by Hisli. The BDI is a 21-item, 4-point Likert-type scale (0–3 points) used to measure the presence and severity of depressive symptoms. The total score obtained from the scale varies between 0–63. Depression severity is classified as minimal (0–9), mild (10–16), moderate (17–29) and severe (30–63) according to total scores. Higher scores indicate more severe depressive symptoms. The Cronbach's alpha reliability coefficient in this study was found to be *α* = 0.92 [23]. This scale has previously been used in fibromyalgia patients, and its validity and reliability have been demonstrated in this population [24].

Anxiety levels were assessed with the Beck Anxiety Inventory (BAI), originally developed by Beck et al. and adapted into Turkish by Ulusoy et al. The BAI is a 4-point Likert-type scale (0–3 points) consisting of a total of 21 items used to measure the presence and severity of anxiety symptoms. The total score obtained from the scale varies between 0 and 63. Total scores are classified according to the severity of anxiety as minimal (0–7), mild (8–15), moderate (16–25) and severe (26–63). Higher scores indicate more severe anxiety symptoms. Cronbach's alpha reliability coefficient in this study was determined as *α* = 0.90 [25]. This scale has also been previously used in studies with fibromyalgia patients, and its validity and utility have been supported in this population [24].

Somatosensory Amplification Scale (SAS) was used to measure perceptual sensitivity towards bodily sensations. The original scale was developed by Barsky et al. and the Turkish validity and reliability study was conducted by Güleç et al. The BPSAS is a 5-point Likert-type scale (1 = strongly disagree, 5 = strongly agree) consisting of a total of 10 items evaluating the tendency of individuals to perceive ordinary bodily sensations as disturbing, intense or harmful. Total scores range from 10 to 50, with higher scores indicating increased perceptual sensitivity to bodily sensations. The Cronbach's alpha reliability coefficient in this study was determined as *α* = 0.84 [26]. The SAS scale has previously been successfully used in FM and other chronic pain patients and its validity has been demonstrated [18].

TDP was assessed with the TDP Scale (DS-14) originally developed by Denollet and adapted into Turkish by Alçelik et al. The scale consists of two subscales of 7 items: negative affectivity and social inhibition. The items are scored on a 5-point Likert scale ranging from 0 (false) to 4 (true). Participants who scored ≥ 10 on both subscales were categorised as having TDP. The Cronbach's alpha reliability coefficient in this study was found to be *α* = 0.88 [27]. This scale has been previously applied in fibromyalgia patients and found to be psychometrically reliable [15].

### 2.3. Statistical Analysis

Statistical analyses were performed using IBM SPSS version 27. Data were considered to follow a normal distribution if the skewness and kurtosis values were within the range of −1.5 to +1.5 [28]. The comparison of categorical variables between groups was conducted using the Chi-square test.

Given that the study included three independent groups, numerical variables with a normal distribution were analyzed using analysis of covariance (ANCOVA), adjusting for relevant covariates, followed by Tukey's post hoc test. Since some sociodemographic variables (e.g., education level, socioeconomic status, employment status, alcohol use, and chronic medical conditions) were found to differ significantly between groups, these were included as covariates in ANCOVA and mediation models where appropriate. For numerical variables that did not follow a normal distribution, the Kruskal–Wallis H test was applied, with Bonferroni correction. Pairwise group comparisons were performed using either the *t*-test or the Mann–Whitney *U* test, depending on the normality of the data.

For correlation analyses, Pearson correlation was applied to normally distributed data, whereas Spearman correlation was used for non-normally distributed data. To assess the mediating effect of somatosensory amplification on the relationship between TDP severity and fibromyalgia severity, mediation analysis was performed using the IBM SPSS Process macro. A regression model based on the bootstrap method with 5000 resamples was employed. A *p* value of < 0.05 was considered statistically significant for all analyses.

## 3. Results

A total of 159 individuals participated in our study in three groups as FM patients with TDP (F + D+) (*n*: 56), FM patients without TDP (F + D−) (*n*: 48), and HC (*n*: 55). The mean ages of these groups were 45.93 ± 11.01, 49.17 ± 11.18, 46.60 ± 9.64, respectively, and they had similar gender distributions. The disease durations of those in the FM group were 11.07 ± 7.95, 9.57 ± 6.43, respectively, and were approximately similar.

The frequency of history of psychiatric illness and familial history of psychiatric and familial fibromyalgia was significantly higher in the F+D+ group. The HC group differed from both FM groups in terms of sociodemographic characteristics. The demographic and clinical characteristics of the participants are presented in detail in Table 1.

The depression, anxiety and SAS scores of the F+D+ group were statistically significantly higher than the F + D− and HC groups (*p* < 0.001). Post hoc analyses showed that these significant differences were mainly due to the higher scores in the F+D+ group. In addition, it was determined that the scores of the WPI and SS were significantly higher in patients with FM than in those without TDP. It is shown in detail in Table 2.

In the correlation analyses, significant positive correlations were found between the total and subtypes of TDP scale scores and depression (*r*: 0.794, *p* < 0.001), anxiety (*r*: 0.729, *p* < 0.001) and SAS (*r*: 0.642, *p* < 0.001), WPI and SS scores. It is shown in Table 3 in detail.

**Figure 1:**
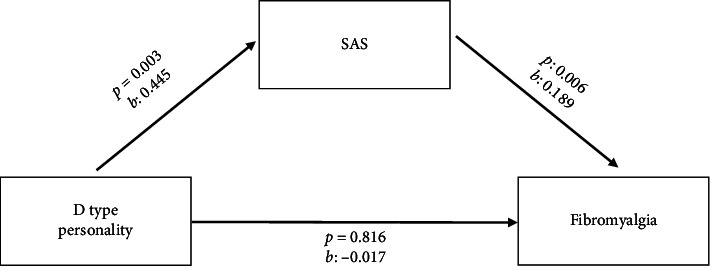
Mediation model of the relationship between TDP and fibromyalgia. Direct effect: *b* = −0.101, *p*=0.188 (not statistically significant). Indirect effect: *b* = 0.084, 95% CI [0.018, 0.172] (statistically significant) *b*: unstandardised beta coefficient, *p*: significance level.

The model created to evaluate the mediating effect of exaggerating body sensations in the relationship between TDP severity and FM severity in the F+D+ group is presented in Figure 1.

This mediation model was found to be statistically significant (*p*: 0.023). It was also found to explain approximately 15% of the total variance in FM severity (*R*^2^: 0.151). As seen in detail in Figure 1, the total effect (*b*: −0.017, 95% Confidence Interval [CI]: −0.167 to 0.132) and direct effect (*b*: −0.101, 95% CI: −0.254 to 0.051) of TDP severity on FM severity were not statistically significant. However, the indirect effect of TDP severity on FM severity was mediated through exaggeration of body sensations and was statistically significant (*b*: 0.084, 95% CI: 0.018 to 0.172).

The fully standardised effect size (*K*^2^) was 0.15, indicating a moderate mediation effect. These findings suggest that exaggeration of body sensations plays a significant mediating role in the relationship between TDP and fibromyalgia severity.

## 4. Discussion

The main aim of this study was to investigate the relationship between TDP traits and depression, anxiety and somatosensory amplification in FM patients and to evaluate the mediating role of somatosensory amplification in the relationship between TDP and FM severity.

This study examined the influence of TDP traits on depression, anxiety, and the tendency to increase bodily sensations in individuals diagnosed with FM. The findings show that the F+D+ group exhibited high levels of depression, anxiety, and heightened somatosensory perception compared to the F + D− and HC group. Furthermore, the prevalence of past psychiatric history was significantly more prevalent in the F+D+ group.

Our correlation analyses showed a positive relationship between TDP and FM severity. Moreover, somatosensory amplification was identified as a mediating mechanism between TDP and FM severity. Although the groups showed significant differences in sociodemographic variables such as education level, employment status, and socioeconomic status, these factors were statistically controlled in the ANCOVA and mediation models. The main findings regarding the relationship between type D personality, depression, anxiety, and somatosensory amplification remained significant after these adjustments, which strengthens the internal validity of the study.

The frequency of psychiatric history was found to be higher in FM patients with TDP. This is similar to previous studies showing the relationship between TDP and psychiatric factors such as depression and anxiety [4, 29]. This relationship may be explained by the fact that both negative affect and social inhibition predispose to psychiatric disorders.

Research has shown a significant relationship between TDP and the severity of anxiety and depression in FM patients. Negative affectivity and social inhibition, key features of TDP, reduce psychological resilience and lead to inadequate coping mechanisms in stressful situations. Individuals with high negative affectivity are significantly more likely to develop depressive and anxiety symptoms. This has been consistently shown in FM patients [9, 30]. Similar to this finding, previous research has reported that TDP traits are associated with depression and anxiety severity in different clinical populations. One study investigated the relationship between TDP and premenstrual syndrome (PMS) and showed that individuals with PMS had significantly higher levels of depression and TDP traits compared to the control group [31]. In our study, strong positive correlations were found between TDP and depression and anxiety symptoms, which are consistent with previous studies.

In studies, the relationship between TDP and FM pain severity, WPI and SS was found to be clinically significant. In a study, higher pain scores on the Visual Analogue Scale (VAS) were found in FM patients with TDP. In this study, higher scores were strongly associated with negative affect and social inhibition [11]. Our study was similar to these findings, showing significant correlations between social inhibition and WPI and FM SS scores. This highlights the potential influence of TDP traits on pain perception and symptom burden. Previous studies have reported more severe fibromyalgia symptoms in individuals with high social inhibition, which is consistent with the findings of our study [15].

Social inhibition, a component of TDP, may be directly related to social withdrawal in FM patients. At the same time, individuals with high negative affectivity have difficulty expressing their emotions and are prone to develop social isolation. FM patients often avoid social activities due to chronic pain. The presence of TDP may worsen this situation and lead to a lack of social support [11, 32].

In addition to personality traits and psychological factors, genetic predisposition and familial factors play an important role in the development of FM. From this point of view, in the guideline titled ‘Fibromyalgia Diagnosis and Treatment Recommendations' published by the Turkish Physical Medicine and Rehabilitation Association and in many studies, family history is stated as an important risk factor for FM [33, 34]. This suggests that genetic or familial predisposition may play a role in the occurrence of FM. Previous studies have also shown that FM shows a familial predisposition, supporting that the disease may have a genetic basis [35]. Additionally, twin studies have demonstrated that TDP has an estimated genetic component of approximately 40% [36]. Our study provides data on the higher prevalence of a history of psychiatric disorders in the families of FM patients with TDP traits. Additionally, the presence of a family history of psychiatric disorders is thought to influence children's emotion regulation skills, contributing to the development of negative affectivity and social inhibition in later life. In individuals raised in an anxious and stressful family environment, inadequate coping mechanisms that develop at an early stage may reinforce negative affectivity and avoidance behaviors. The literature has shown that social withdrawal and avoidance behaviours are frequently observed in children of individuals with high levels of social inhibition [37].

Our study showed that the effect of TDP on FM severity was not direct, but was mediated by the tendency to increase bodily sensations. Individuals with TDP tend to perceive bodily sensations as more intense and more threatening. This leads to more severe FM symptoms. Negative affect has been shown to increase pain perception and therefore increase SS. In addition, negative affect may contribute to social inhibition, limiting access to social support and contributing to psychological burden. This can potentially worsen disease severity. Previous studies have reported that FM patients show significantly higher levels of negative affect and somatosensory amplification than healthy controls [9, 38]. A study investigated the increase in bodily sensations and the severity of FM. They found that sensitivity to somatic symptoms was high in FM patients. It has been reported that this may lead to an increase in disease burden [38]. However, the psychosocial factors causing this discrepancy have not been fully defined. Our study suggests that TDP traits may play an important role in FM severity. Previous studies have indicated that impairments in emotion regulation skills may cause increased pain perception in FM patients [39]. However, the specific relationship and influencing factors between this disorder and TDP traits have not been adequately investigated. Our findings clearly linked somatosensory amplification with TDP. Our study extends this literature and highlights an important psychological mechanism contributing to FM and SS.

In a study on individuals with coronary artery disease, the effect of TDP on health behaviours was examined. TDP was reported to be associated with more severe perception of physical symptoms [10]. Similarly, studies have examined the relationship between TDP, physical symptoms and subjective perception of health. The findings showed a significant relationship between TDP and the tendency to magnify bodily sensations [40]. Our study found that TDP showed similar characteristics in FM patients. However, TDP has not been either explicitly identified or considered as an independent variable in previous research. This suggests that TDP may have been overlooked as an important cause of negative affectivity and the tendency to increase bodily sensations in previous studies. Our study aims to fill this gap by showing how TDP influences disease severity in FM patients through both psychological (negative affect, social inhibition) and physiological (pain perception, somatosensory amplification) components.

Our study showed that the effect of TDP on FM severity is not direct, but occurs indirectly through a tendency to exaggerate bodily sensations. Somatosensory amplification increases pain sensitivity and lowers pain threshold by increasing bodily perception. This process is associated with a central sensitisation mechanism and may lead to increased SS. Furthermore, TDP increases levels of anxiety and depression, negative emotional responses and maladaptive coping mechanisms such as pain catastrophising. This process can progressively worsen the pain sensation in individuals [40, 41]. Therefore, the tendency to exaggerate bodily sensations in patients with TDP may be an important psychosocial factor providing the link between increased psychological vulnerability to negative emotions and stress and the severity of FM symptoms.

### 4.1. Limitations

The cross-sectional case-control design of this study is insufficient to determine whether TDP plays a causal role in the development of FM. Although our study demonstrated an association between TDP and SS in FM patients, longitudinal studies are needed to clarify the direction of this relationship over time.

Furthermore, the fact that the study was conducted in a tertiary health centre may have led to an overrepresentation of patients with more severe symptoms. This may limit the generalisability of the findings to patients with milder symptoms or community-based populations.

The psychiatric rating scales used in this study are self-report-based; participants' responses may have been influenced by individual perceptions. Given the potential risk of response bias, future research may benefit from the inclusion of objective biological measures such as functional magnetic resonance imaging (fMRI), quantitative sensory testing (QST), electrophysiological measures, or markers of inflammation to strengthen the validity of the findings. These measures could objectively support the association of TDP and somatosensory amplification with FM symptoms.

Psychiatric comorbidities were not completely excluded. Although the psychiatric histories of the participants were recorded, the influence of conditions such as anxiety and depression on the relationship between TDP and FM could not be fully isolated. In future studies, it is recommended that participants with more homogeneous psychiatric diagnoses be selected and these variables be examined in more detail in order to overcome this limitation.

Although education level, socioeconomic status, alcohol consumption, presence of chronic diseases and employment status were found to be statistically significant factors in our study, these variables were not included in the discussion section since they were not included in the primary study results.

In order to account for potential confounding variables, additional analyses were conducted using ANCOVA, where sociodemographic factors that significantly differed between groups were included as covariates. However, in some comparisons where the assumption of normality was not met and nonparametric tests such as the Kruskal–Wallis test were applied, it was not possible to statistically control for potential confounding variables. This constitutes a methodological limitation and should be considered when interpreting those specific findings.

Finally, increasing the sample size and including different sociodemographic groups may increase the generalisability and reliability of the findings.

## 5. Conclusion

Our study is one of the limited number of studies examining the effect of TDP on depression, anxiety levels and somatosensory amplification in FM patients. Our findings showed that FM patients with TDP had significantly higher levels of depression, anxiety and somatosensory amplification than FM patients without TDP and healthy controls. We also found that somatosensory amplification mediated the relationship between TDP and FM severity.

Considering these findings together, it can be hypothesised that it is necessary to include psychosocial interventions in the treatment plans of FM patients with TDP and a high tendency to exaggerate bodily sensations. Cognitive restructuring enables negative thoughts to evolve into more realistic and constructive thoughts. In these patients, cognitive restructuring may reduce the tendency to catastrophise bodily sensations. Psychoeducation may play an important role in the relief of FM and related symptoms. Psychoeducation can help people better understand their symptoms, reduce anxiety and improve treatment compliance. Furthermore, mindfulness-based therapies can increase patients' awareness of bodily sensations. With these therapies, they can assess their pain more objectively and cope with their symptoms more effectively. Such interventions can reduce FM patients' perception of pain. It may also improve symptom management and positively affect their quality of life.

Our results emphasise the necessity of a multidisciplinary approach in FM management. It draws attention to the importance of psychosocial factors in the disease course. Identification of TDP as a potential risk factor in FM patients may help to develop personalised and comprehensive treatment strategies. In future research, it would be useful to directly test the effectiveness of psychosocial interventions such as cognitive restructuring, psychoeducation and mindfulness-based therapies in FM patients.

## Figures and Tables

**Table 1:** Comparison of sociodemographic characteristics of participants.

	**F + D+ (*n* = 56)**	**F + D− (*n* = 48)**	**HC (*n* = 55)**			
	**Mean ± standard deviation**			**Statistics**	**p**	**Post hoc**

Age (years)	45.93 ± 11.01	49.17 ± 11.18	46.60 ± 9.64	*F* = 1.304	0.274	
Duration of fibromyalgia	11.07 ± 7.95	9.57 ± 6.43	—	*t* = 1.031	0.305	

	***n* (%)**					

Gender				*X* ^2^ = 3.042	0.218	
Female	51 (91.1%)	39 (81.3%)	44 (80%)			
Male	5 (8.9%)	9 (18.8%)	11 (20%)			
Location				*X* ^2^ = 2.380	0.304	
Urban center	36 (64.3%)	31 (64.6%)	42 (76.4%)			
District-village	20 (35.7%)	17 (35.4%)	13 (23.6%)			
Marital status				*X* ^2^ = 4.060	0.131	
Married	46 (82.1%)	36 (75%)	36 (65.5%)			
Single	10 (17.9%)	12 (25%)	19 (34.5%)			
Education level				*X* ^2^ = 24.156	**< 0.001**	**HC > D+ = D-**
Primary school	22 (39.3%)	28 (58.3%)	7 (12.7%)			
Middle school	7 (12.5%)	4 (8.3%)	9 (16.4%)			
High school	14 (25%)	10 (20.8%)	20 (36.5%)			
University	13 (23.2%)	6 (12.5%)	19 (34.5%)			
Employment status				*X* ^2^ = 23.968	**< 0.001**	**HC > D+ = D-**
Employed	16 (28.6%)	14 (29.2%)	36 (65.5%)			
Retired	11 (19.6%)	13 (27.1%)	11 (20%)			
Unemployed	29 (51.8%)	21 (43.8%)	8 (14.5%)			
Socioeconomic status				*X* ^2^ = 19.989	**< 0.001**	**HC > D+ = D−**
Poor	15 (26.8%)	11 (22.9%)	1 (1.8%)			
Moderate	32 (57.1%)	23 (47.9%)	29 (52.7%)			
Good	9 (16.1%)	14 (29.2%)	25 (45.5%)			
Smoking				*X* ^2^ = 1.110	0.574	
Yes	18 (32.1%)	11 (22.9%)	16 (29.1%)			
No	38 (67.9%)	37 (77.1%)	39 (70.9%)			
Alcohol consumption				*X* ^2^ = 7.880	**0.019**	**HC > D+ = D−**
Yes	3 (5.4%)	6 (12.5%)	13 (23.6%)			
No	53 (94.6%)	42 (87.5%)	42 (76.4%)			
Chronic medical condition				*X* ^2^ ^2^ = 6.584	**0.037**	**D+ = D− > HC**
Yes	27 (48.2%)	25 (52.1%)	16 (29.1%)			
No	29 (51.8%)	23 (47.9%)	39 (70.9%)			
Fibromyalgia medication use				*X* ^2^ = 1.145	0.285	
Yes	22 (40.7%)	14 (30.4%)	—			
No	32 (34.6%)	32 (69.6%)	—			
History of psychiatric illness				*X* ^2^ = 13.205	**0.001**	**D+ > D− = HC**
Yes	29 (51.8%)	14 (29.2%)	11 (20%)			
No	27 (48.2%)	34 (70.8%)	44 (80%)			
Family history of psychiatric disorders				*X* ^2^ = 11.124	**0.004**	**D+ > D− = HC**
Yes	29 (51.8%)	10 (20.8%)	18 (32.7%)			
No	27 (48.2%)	38 (79.2%)	37 (67.3%)			
Family history of fibromyalgia				*X* ^2^ = 18.253	**< 0.001**	**D+ > D− = HC**
Yes	25 (44.6%)	5 (10.4%)	10 (18.2%)			
No	31 (55.4%)	43 (89.6%)	45 (81.8%)			

*Note:p*: significance level, *X*^2^: Chi-square test statistic. F + D+: fibromyalgia with type D personality, F + D−: fibromyalgia without type D personality.

Abbreviations: HC, healthy controls; SD, standard deviation.

**Table 2:** Comparison of clinical variables between groups.

	F + D+ (*n* = 56)	F + D- (*n* = 48)	HC (*n* = 55)	Statistics	Post hoc
	Mean ± SD or median (Q1–Q3)				
Total score of the fibromyalgia scale	22.66 ± 4.2	17.85 ± 2.95	—	*F* = 26.490^∗a^	
Widespread pain score	13.38 ± 2.91	11.23 ± 2.22	—	*F* = 13.875^∗∗a^	
Symptom severity score	9.5 (8–11)	6 (5–8)	—	*U* = 467.500^∗^	
SAS	31.88 ± 8.77	20.6 ± 8.93	23.73 ± 7.53	*F* = 17.007^∗b^	F + D+ > F + D− = HC
BAI	36.5 (26.5–42)	12 (7–19.75)	5 (2–11)	KW = 102.719^∗^	F + D+ > F + D− > HC
BDI	31.84 ± 9.88	10.73 ± 6.78	6.42 ± 5.52	*F* = 119.979^∗b^	F + D+ > F + D− > HC
DS-14	39 (34–44.75)	7 (5–10.75)	9 (5–13)	KW = 107.610^∗^	F + D+ > F + D− = HC
Negative affectivity	17.36 ± 5.01	3.15 ± 2.46	5.16 ± 3.58	*F* = 153.067^∗b^	F + D+ > HC > F + D−
Social inhibition	22 (18.5–24)	4 (3–7)	4 (2–7)	KW = 105.803^∗^	F + D+ > F + D− = HC

*Note: n*: number of samples, *p*: significance level, Q1: first quartile, Q3: third quartile, DS-14: type D personality Scale. F + D+: fibromyalgia with type D personality, F + D−: fibromyalgia without type D personality, *t*: Student *T* test, U: Mann–Whitney *U* test, KW: Kruskal–Wallis test.

Abbreviations: BAI, Beck Anxiety Inventory; BDI, Beck Depression Inventory; HC, healthy controls; SAS, Somatosensory Amplification Scale; SD, standard deviation.

^a^Adjusted for history of psychiatric illness, family history of psychiatric disorders, and family history of fibromyalgia.

^b^Adjusted for education level, employment status, socioeconomic status, alcohol consumption, chronic medical condition, history of psychiatric illness, family history of psychiatric disorders, and family history of fibromyalgia.

^∗^ *p* < 0.001.

^∗∗^ *p* < 0.05.

**Table 3:** Correlation between DS-14 scale scores and clinical variables.

	DS-14		Negative affectivity		Social inhibition	
	*r*	*p*	*r*	*p*	*r*	*p*
Total score of the fibromyalgia scale	0.509	< 0.001	0.465	< 0.001	0.515	**< 0.001**
Widespread pain score	0.304	0.002	0.255	0.009	0.328	**< 0.001**
Symptom severity score	0.576	< 0.001	0.554	< 0.001	0.558	**< 0.001**
SAS	0.580	< 0.001	0.540	< 0.001	0.578	**< 0.001**
BAI	0.729	< 0.001	0.681	< 0.001	0.723	**< 0.001**
BDI	0.794	< 0.001	0.734	< 0.001	0.796	**< 0.001**

*Note:p*: significance level, *r*: correlation coefficient, DS-14: type D personality Scale. Level of Correlation Coefficient: (i) 0 < *r* < 0.299: weak positive. (ii) 0.3 < *r* < 0.599: moderate positive. (iii) 0.6 < *r* < 0.799: strong positive. (iv) 0.8 < *r* < 0.999: very strong positive.

Abbreviations: BAI, Beck Anxiety Inventory; BDI, Beck Depression Inventory; SAS: Somatosensory Amplification Scale.

## Data Availability

The data supporting the findings of this study are available from the corresponding author upon reasonable request. The data are not publicly available due to privacy or ethical restrictions.
